# Efficacy of idecabtagene vicleucel in patients with relapsed/refractory multiple myeloma and prior central nervous system manifestation: A multicenter real‐world analysis

**DOI:** 10.1002/hem3.70192

**Published:** 2025-08-18

**Authors:** Markus Maulhardt, Simon Call, Hristo Boyadzhiev, Anca Maria Albici, Keven Hörster, Amelie Boquoi, Snjezana Janjetovic, Anna Ossami Saidy, Marcel Teichert, Annamaria Brioli, Christian Schultze‐Florey, Florian Heidel, Philipp Schindler, Natalie Schub, Enver Aydilek, Matthias Stelljes, Michael Daskalakis, Carolin Krekeler, Justin Hasenkamp, Cyrus Khandanpour, Ulrike Bacher, Hans Christian Reinhardt, Georg Lenz, Friedrich Stölzel, Thomas Pabst, Bastian von Tresckow, Gerald Wulf, Philipp Berning, Evgenii Shumilov

**Affiliations:** ^1^ Department of Hematology and Medical Oncology University Hospital Goettingen Goettingen Germany; ^2^ Department of Medicine A, Hematology, Oncology and Pneumology University Hospital Muenster Muenster Germany; ^3^ Department of Medical Oncology, Inselspital, University Hospital Bern University of Bern Bern Switzerland; ^4^ Habichtswald Hospital Kassel Germany; ^5^ Division of Stem Cell Transplantation and Cellular Immunotherapies University Hospital Schleswig‐Holstein Kiel Germany; ^6^ Department of Hematology and Stem Cell Transplantation, West German Cancer Center and German Cancer Consortium (DKTK Partner Site Essen) University Hospital Essen, University of Duisburg‐Essen Essen Germany; ^7^ Department of Hematology and Cell Therapy Helios Klinikum Berlin‐Buch Berlin Germany; ^8^ Department of Hematology, Hemostasis, Oncology and Stem Cell Transplantation Hannover Medical School Hannover Germany; ^9^ Clinic for Radiology, Faculty of Medicine Muenster University Muenster Germany; ^10^ Evangelisches Klinikum Bethel University Hospital Bielefeld Bielefeld Germany; ^11^ Department of Hematology and Central Hematology Laboratory Inselspital, Bern University Hospital, University of Bern Bern Switzerland; ^12^ Department of Hematology and Oncology University Hospital Schleswig‐Holstein, University of Luebeck Luebeck Germany

Central nervous system (CNS) involvement in multiple myeloma (MM) is a rare complication associated with poor prognosis and a median overall survival (mOS) between 2 and 7 months.^1^, ^2^ CNS involvement is characterized by plasma cell infiltration of the CNS parenchyma, meninges, or cerebrospinal fluid (CSF).^1^, ^3^ B‐cell maturation antigen (BCMA)‐directed chimeric antigen receptor (CAR) T‐cell therapies have significantly improved the treatment options for patients with relapsed/refractory (r/r) MM.^4^, ^5^ Currently, two CAR T‐cell products have been approved for r/r MM, ide‐cel (idecabtagene vicleucel) and cilta‐cel (ciltacabtagene autoleucel). With ide‐cel being the first approved CAR T‐cell therapy in r/r MM, it offers the longest clinical experience to date.^4^ Previous experience with CD19‐directed CAR T‐cells in patients (pts) with r/r large B‐cell lymphomas and CNS involvement has demonstrated their ability to cross the blood–brain barrier, as well as to expand and persist in CNS compartments.^6^, ^7^, ^8^ In contrast, for r/r MM pts with a history of CNS disease (MM‐CNS), real‐world evidence with respect to the efficacy and safety profiles of CAR T‐cells remains limited, as these pts were excluded from the pivotal studies.^4^, ^5^ To address this gap, we sought to evaluate the efficacy and toxicity profiles of BCMA‐directed CAR T‐cell therapy in MM‐CNS pts in a real‐world context.

We conducted a multicenter retrospective study including r/r MM pts undergoing ide‐cel treatment between March 2022 and May 2024 at seven German/Swiss tertiary care centers. Pts were grouped by the presence of CNS disease before ide‐cel infusion. Only pts with intradural and/or intraparenchymal lesions by magnetic resonance imaging (MRI)/computed tomography (CT) brain/spine or detection of myeloma cells in the CSF were regarded as MM‐CNS pts. Descriptive and survival analyses, including propensity score matching (PSM) between MM‐CNS and non‐MM‐CNS pts (optimal matching with 1:3 ratio; age at ide‐cel, number of prior therapy lines, and IMWG response at ide‐cel as covariates), were performed. Clinical data were gathered from the medical records.

Before CAR T‐cell infusion, all patients received lymphodepleting chemotherapy with fludarabine and cyclophosphamide in accordance with guideline recommendations. Grading of cytokine release syndrome (CRS) and immune effector cell‐associated neurotoxicity syndrome (ICANS) was performed according to the American Society for Transplantation and Cellular Therapy consensus grading for CRS and ICANS.^9^ The CNS response to CAR T‐cell therapy was classified as complete remission (CR), partial remission (PR), stable disease (SD), and progressive disease (PD). The serologic response was assessed according to the established IMWG criteria.^10^ The toxicity management guidelines were consistently applied for all pts, irrespective of the presence of CNS manifestations.

This study was approved by the Institutional Review Board of the University of Muenster (2024‐068‐f‐S). Overall survival (OS) was calculated as the time from CAR T‐cell infusion to death or last follow‐up (FU). Progression‐free survival (PFS) was calculated as the time from CAR T‐cell infusion to disease progression, death, or last FU.

In total, 150 r/r MM patients underwent ide‐cel therapy in the participating study centers during the study period. Ten (6.7%) pts from five centers met the criteria for CNS disease before CAR‐T treatment. Median time from diagnosis to ide‐cel infusion for MM‐CNS pts was 5.6 years (range: 3.6–13.6), and median time from leukocyte apheresis to CAR‐T treatment was 61 days (range: 47–112).

The median age of MM‐CNS pts at CAR‐T infusion and the median number of therapy lines before ide‐cel were 61 years (range: 47–71 years) and 5 (range: 2–8), respectively. Triple‐ and penta‐refractoriness was documented in 9/10 and 7/10 of the patients, respectively. All patients developed secondary CNS involvement during the disease course, after a median of 3 (range: 1–7) therapy lines. CNS manifestation was detected in one patient during bridging therapy 2 weeks before ide‐cel.

CNS manifestations were characterized by parenchymal lesions (5/10) and/or leptomeningeal manifestations (6/10). CNS myeloma was diagnosed by MRI (3/10), CT (1/10), both MRI and CT (4/10), or a combination of imaging and CSF diagnostics (2/10) (Supporting Information S1: Table 1). Before ide‐cel infusion, seven pts received CNS‐penetrating systemic myeloma therapies, including immunomodulatory drug (IMiD)‐containing regimens (5 pts), anti‐CD38 antibody treatments (5 pts), and MTX‐based therapies (2 pts). Additionally, radiation therapy was applied in 3/10 cases. Intrathecal therapy was administered to 4/10 of MM‐CNS pts. Two pts received a combination of radiation and surgery (Table 1). Supporting Information S1: Table 2 shows applied specific treatments and outcomes of the 10 MM‐CNS pts in detail.

**Table 1:** Outcomes and toxicities of central nervous system (CNS) myeloma patients.

Parameters	MM‐CNS pts (*N* = 10)
CNS‐directed treatments	
Chemotherapy‐based	3
Conventional MM regimens + radiation	4
Intrathecal therapy	4
Consolidation with HDCT/ASCT	1
Surgery plus radiation	2
Bridging therapy	
Conventional MM regimens	5
Chemotherapy‐based	4
Radiotherapy	1
Serologic response at ide‐cel infusion	
CR	3
(VG)PR	5
SD/PD	2
CNS evaluation at ide‐cel infusion	
Imaging only	5
Cranial CT only	1
MRI brain and spine	3
MRI brain *and* CSF	2
CSF only	1
Clinical assessment only	2
CNS response at ide‐cel infusion	
CR	2
PR	6
SD	0
PD	2
CRS	
None	1
Grade 1	3
Grade 2	4
Grade 3	2
Grade 4	0
ICANS	
None	8
Grade 1	2
Steroids applied for CRS/ICANS	8
Tocilizumab applied for CRS/ICANS	8
Best serologic response after ide‐cel	
CR	4
PR	5
SD	0
PD	1
CNS response evaluation after ide‐cel	
Imaging only	7
cCT	0
cMRI	6
cCT *and* cMRI	1
Imaging *and* CSF	3
Best response of CNS myeloma after ide‐cel	
CR	6
PR	2
SD	1
PD	1
Relapse MM after ide‐cel in total	4
CNS relapse/progress after ide‐cel	2

Abbreviations: cCT, cranial computed tomography; cMRI, cranial magnetic resonance imaging; CR, complete remission; CRS, cytokine release syndrome; CSF, cerebrospinal fluid; HDCT/ASCT, high‐dose chemotherapy and autologous stem cell transplantation; ICANS, immune effector cell‐associated neurotoxicity syndrome; ide‐cel, idecabtagene vicleucel; MM, multiple myeloma; PD, progressive disease; PR, partial remission; pts, patients; SD, stable disease; VGPR, very good partial remission.

At the time of ide‐cel treatment, CR in CNS was documented in 2/10 of pts, 6/10 had a PR, and 2/10 had PD as assessed by MRI/CT brain/spine with or without CSF diagnostics. Serologic response rates at ide‐cel infusion were CR in 3/10, VGPR/PR in 5/10, and PD in 2/10 pts (Table 1, Supporting Information S1: Table 2).

At first response assessment one month post‐CAR‐T, best serologic responses were as follows: 4/10 CR, 5/10 pts with VGPR/PR, and 1/10 in PD. Regarding CNS disease, 3/10 pts maintained response until last FU (CR: 2, PR: 1), whereas 5/10 pts improved response to CR (4/10) and PR (1/10), respectively. In the two remaining pts, SD (1/10) or PD (1/10) as best response was documented post‐ide‐cel (Table 1; Figure 1A). Information on CAR T‐cell persistence and CSF plasma cell clearance was available in one patient (Patient 8). T cells were detected in CSF taken 30 days post‐infusion with approximately 2.8 T cells/µL and 17.9% CAR expression (Supporting Information S2: Figure 1). Along this line, this patient achieved a CR of the spinal CNS manifestation coming from a PR, as evaluated by MRI, along with a serological CR (Figure 1A; Supporting Information S2: Figure 1). Overall, 4/10 pts experienced relapse post‐CAR‐T. Among these, two pts showed PD/relapse of the CNS disease and serologic relapse (pts #6 and 9), while the remaining two pts (#7 and 5) developed serologic PD/relapse only (Figure 1A).

**Figure 1:**
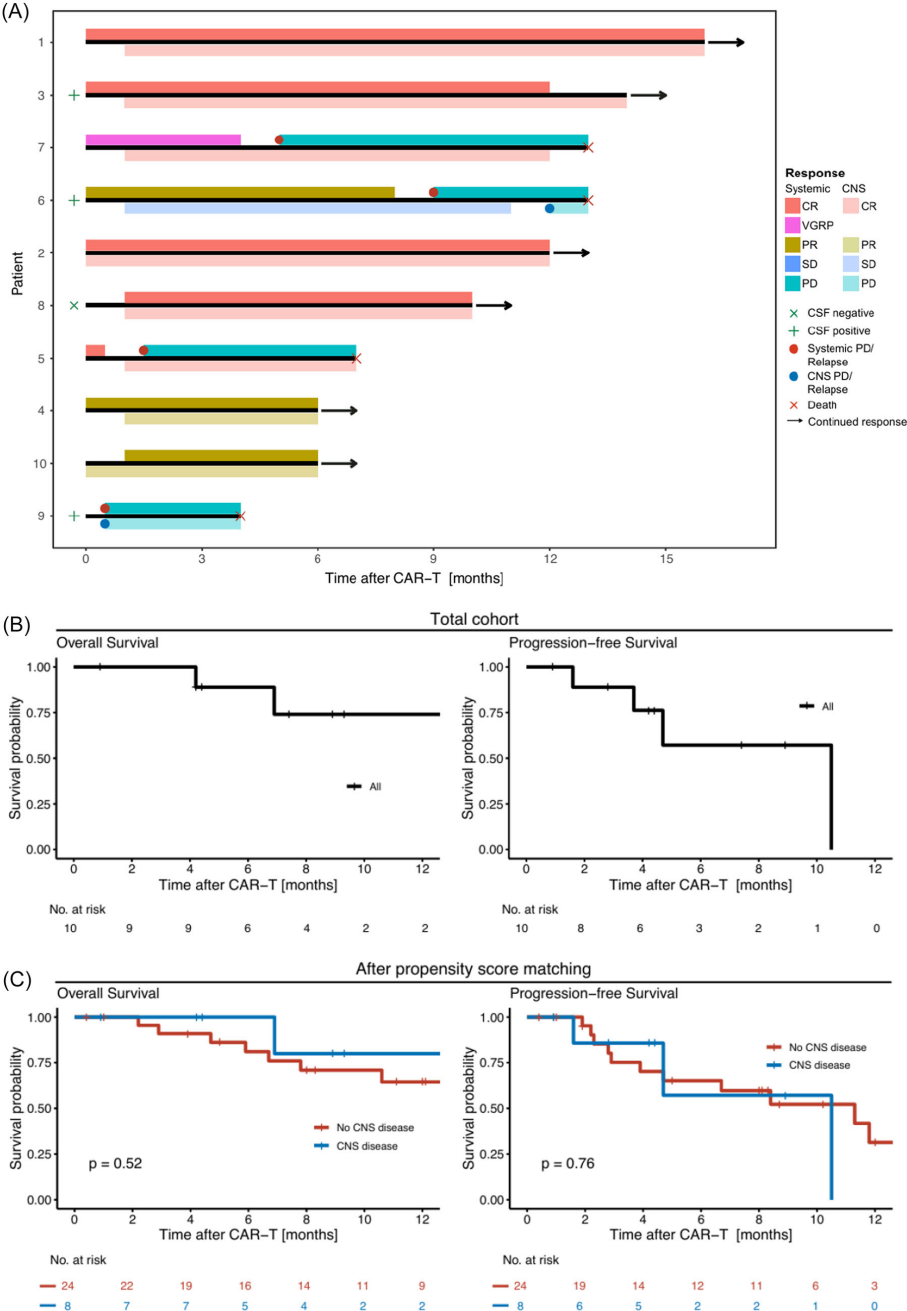
**Clinical courses after treatment with ide‐cel (idecabtagene vicleucel). (A)** Swimmer plot depicting clinical courses after chimeric antigen receptor (CAR)‐T treatment for all 10 patients, with systemic (upper line) and central nervous system (CNS)‐specific disease status (lower line) shown for each patient separately. With respect to subsequent therapies after CAR‐T failure, Patient 5 was treated with talquetamab in systemic relapse, whereas Patient 7 received salvage therapies with bortezomib, dexamethasone, cisplatin, doxorubicin, cyclophosphamide, etoposide (VD‐PACE), teclistamab, epirubicin, etoposide, ifosfamide (IEV), and allogeneic stem cell transplantation (alloSCT). Patient 6 received a stem cell boost and salvage therapy with pomalidomide, dexamethasone, and selinexor after CAR‐T failure. Patient 9 opted for best supportive care following refractory disease post‐CAR‐T and declined any further therapy. **(B)** Kaplan–Meier estimates for patients (pts) with confirmed CNS myeloma showing overall survival (OS) and progression‐free survival (PFS). **(C)** OS and PFS after propensity score matching with a matched cohort of 24 pts without CNS myeloma. Abbreviations: CR, complete remission; CSF, cerebrospinal fluid; PD, progressive disease; PR, partial remission; SD, stable disease; VGPR, very good partial remission.

Figure 1B depicts Kaplan–Meier estimates for all MM‐CNS pts. With a median FU of survivors of 11 months, a median OS of 12.9 months and a median PFS of 10.5 months were observed. To compare outcomes between pts with and without CNS manifestations, we applied PSM, identifying a matched cohort of 24 pts without CNS myeloma. After matching, survival outcomes and serologic response rates were comparable for the MM‐CNS cohort and non‐CNS cohort (median OS: 13 months [MM‐CNS] vs. not reached [non‐CNS myeloma], P = 0.52; median PFS: 10.5 vs. 11.3 months, P = 0.76) (Figure 1C). Overall response rates (CR/VGPR/PR) were 75% for both MM‐CNS and non‐MM‐CNS pts (P = 1.00) (Supporting Information S1: Table 3). Nine patients experienced CRS, of which 3 pts (3/9) had CRS Grade 1, 4 pts (4/9) Grade 2, and 2 pts (2/9) Grade 3. Only 2 pts (2/10) developed ICANS, both Grade 1 (Table 1). Of note, no high‐grade (3‐4) ICANS was documented among MM‐CNS patients, which was consistent with the matched cohort of non‐CNS pts (0/24) (Supporting Information S1: Table 4). All four deaths occurred due to r/r disease (Figure 1A).

This real‐world analysis evaluates the safety and efficacy of BCMA‐directed CAR T‐ cell therapy in MM pts with CNS disease. There were three main findings. First, we observed that CAR T‐cell therapy achieves encouraging response rates in MM‐CNS pts, with 8/10 patients showing continued CNS response. Second, our results suggest that outcomes of CAR T‐cell therapy in a MM‐CNS patient cohort appear to be comparable to a matched cohort of classical non‐CNS myeloma pts after CAR‐T ide‐cel treatment. Nevertheless, statistical non‐significance as well as the low number of patients analyzed do not necessarily imply equivalence, and larger studies are needed to validate these findings. Finally, BCMA‐directed T‐cell lymphocytes measurement in the CSF was performed in one patient, aligning with the rapid clinical and documented radiologic response and confirming their ability to penetrate the blood–brain barrier as well.

To the best of our knowledge, despite the small patient number, our study represents the largest MM‐CNS cohort specifically treated with ide‐cel and the largest cohort diagnosed with CNS myeloma before CAR‐T treatment.^11^ The available data on post‐CAR‐T outcomes for MM with CNS disease are limited to smaller case reports, mainly reporting single cases and small retrospective studies.^12^, ^13^ Although CNS manifestations generally represent a rare complication affecting less than 1% of MM pts, we observed a comparatively high frequency of CNS disease of 6.7% for our cohort.^1^, ^3^ This may be attributable to improved therapies with subsequent clonal evolution/selection of myeloma cells becoming prone to CNS invasion. The improved survival of MM pts is expected to lead to an increased incidence of CNS myeloma as well.^1^

Conventional approaches to treat CNS myeloma include radiotherapy, intrathecal and/or systemic chemotherapy, IMiDs, and anti‐CD38 directed agents, such as daratumumab, which are capable of crossing the blood–brain barrier.^13^, ^14^, ^15^ Although being effective in some CNS myeloma cases, the response duration of these agents is often limited to weeks or a few months only.^13^ These treatment approaches are unlikely to achieve long‐term remissions, highlighting the potential role of CAR T‐cells as a consolidation strategy in this setting.

An improvement of CNS response after ide‐cel was documented in 6 out of 8 (75%) of our pts with active CNS disease, defined as non‐CR status at CAR‐T. Specifically, two pts with PD of CNS myeloma at the start of ide‐cel achieved disease control, presenting with either PR or SD at the first FU. Gaballa et al. reported a 100% CNS response rate by Day 90 post‐CAR‐T.^11^ We also documented high levels of CAR T‐cells at Day 30 in a patient who achieved a rapid response, underscoring the potential persistence of ide‐cel post‐infusion. This aligns with findings by Wang et al. demonstrating detectable levels of BCMA‐directed CAR T‐cells in the CSF of a patient with CNS involvement, with levels peaking at Day 8 after CAR‐T infusion.^12^

In fact, Gaballa et al. recently reported 10 r/r MM pts with CNS disease from five centers treated with either ide‐cel (*n* = 6) or cilta‐cel (*n* = 4), whereas two of these pts were diagnosed with CNS myeloma shortly post‐CAR‐T infusion.^11^ For the 8 pts with confirmed CNS disease before CAR T‐cell infusion, the median OS and PFS were 13.3 and 6.3 months, respectively.^11^ Regarding the development of CAR‐T‐related adverse effects, we and Gaballa et al. both report a few high‐grade CRS/ICANS.^11^ Solely two pts in our cohort had CRS Grade 3, compared to none in the Gaballa cohort. We report no high‐grade ICANS, whereas Gaballa et al. reported one case with ICANS Grade 3.^11^ A strength of our study is the use of PSM with non‐CNS MM patients undergoing ide‐cel treatment, which confirmed comparable survival outcomes. Together, our findings and those of Gaballa et al. highlight encouraging response rates to BCMA‐directed CAR T‐cell therapy among patients with MM and CNS involvement.

However, we acknowledge that other important potential confounders, such as cytogenetic risk profile, performance status, and prior BCMA exposure, were not included in the PSM analysis but could influence outcomes. Additionally, the FU time was relatively limited, thereby reducing the capability to draw long‐term conclusions about the safety and efficacy of CAR T‐cell therapy in patients with CNS myeloma.

Our findings suggest that CAR T‐cell therapy can be effective in MM pts with CNS involvement, improving response with a toxicity profile comparable to non‐CNS pts. Thus, CNS manifestations in r/r MM should not preclude the use of CAR T‐cell treatment for these patients.

## AUTHOR CONTRIBUTIONS

**Markus Maulhardt**: Conceptualization; writing—original draft; investigation; data curation; methodology; formal analysis. **Simon Call**: Investigation; data curation. **Hristo Boyadzhiev**: Investigation; data curation. **Anca Maria Albici**: Investigation; data curation. **Keven Hörster**: Investigation; data curation. **Amelie Boquoi**: Investigation; data curation. **Snjezana Janjetovic**: Investigation; data curation. **Anna Ossami Saidy**: Investigation; data curation. **Marcel Teichert**: Investigation; data curation. **Annamaria Brioli**: Investigation. **Christian Schultze‐Florey**: Investigation. **Florian Heidel**: Investigation; writing—review and editing. **Philipp Schindler**: Investigation; data curation. **Natalie Schub**: Investigation; data curation. **Enver Aydilek**: Investigation; data curation. **Matthias Stelljes**: Investigation; data curation. **Michael Daskalakis**: Investigation; data curation. **Carolin Krekeler**: Investigation. **Justin Hasenkamp**: Investigation. **Cyrus Khandanpour**: Investigation; data curation; writing—review and editing. **Ulrike Bacher**: Investigation; data curation; writing—review and editing. **Hans Christian Reinhardt**: Investigation; writing—review and editing. **Georg Lenz**: Investigation; writing—review and editing. **Friedrich Stölzel**: Investigation; writing—review and editing. **Thomas Pabst**: Investigation; writing—review and editing. **Bastian von Tresckow**: Investigation; writing—review and editing. **Gerald Wulf**: Investigation; writing—review and editing. **Philipp Berning**: Conceptualization; investigation; writing—original draft; supervision; methodology; validation; writing—review and editing; software; data curation. **Evgenii Shumilov**: Conceptualization; investigation; writing—original draft; supervision; methodology; validation; writing—review and editing; data curation.

## CONFLICT OF INTEREST STATEMENT

A.B. has participated in advisory boards from BMS, Janssen, GSK, Sanofi, AstraZeneca, and Menarini; received honoraria from Menarini; and received honoraria and travel support from BMS, Janssen, GSK, Sanofi, AstraZeneca, Amgen, and Takeda. M.S. has served as a consultant for Pfizer, MSD, BMS, Incyte, Takeda, Astellas, and Amgen; as a speaker for Pfizer, Medac, MSD, Astellas, Jazz Pharmaceuticals, Amgen, Novartis, Gilead, Celgene, BMS, AbbVie, and Incyte; has received research funding from Pfizer; and has received travel support from Medac and Pfizer. M.D. received support (travel, accommodations, expenses) from Kite‐Gilead, Novartis, Amgen, and Novo Nordisk and served in a consulting or advisory role for Novartis and Alexion Pharma. G.L. received research grants not related to this manuscript from AGIOS, AQUINOX, AstraZeneca, Bayer, Gilead, Janssen, MorphoSys, Novartis, F. Hoffmann‐La Roche Ltd, and Verastem. G.L. received honoraria not related to this manuscript from ADC Therapeutics, AbbVie, Amgen, AstraZeneca, Bayer, BeiGene, BMS, Celgene, Constellation, Genase, Genmab, Gilead, Hexal/Sandoz, Immagene, Incyte, Janssen, Karyopharm, Lilly, Miltenyi, MorphoSys, MSD, NanoString, Novartis, PentixaPharm, Pierre Fabre, F. Hoffmann‐La Roche Ltd, and SOBI. B.v.T. is an advisor or consultant for Allogene, Amgen, BMS/Celgene, Cerus, Gilead Kite, Incyte, IQVIA, Janssen‐Cilag, Lilly, MSD, Miltenyi, Novartis, Noscendo, Pentixapharm, Pfizer, Pierre Fabre, Qualworld, Regeneron, Roche, SOBI, and Takeda; has received honoraria from AbbVie, AstraZeneca, BMS/Celgene, Gilead Kite, Incyte, Janssen‐Cilag, Lilly, MSD, Novartis, Roche, and Takeda; reports research funding from Esteve (Inst.), MSD (Inst.), Novartis (Inst.), and Takeda (Inst.); reports travel support from AbbVie, AstraZeneca, Gilead Kite, Janssen‐Cilag, Lilly, MSD, Pierre Fabre, Roche, Takeda, and Novartis; and is member of steering committees for Regeneron and Takeda. E.S. received honoraria not related to this manuscript from Gilead, Amgen, Sanofi, Oncopeptides, Stemline, Takeda, Pfizer, BMS, and Lilly.

## FUNDING

Open Access funding enabled and organized by Projekt DEAL.

## Supporting information

Supporting Information.

Supporting Information.

## Data Availability

The data that support the findings of this study are available on request from the corresponding author. The data are not publicly available due to privacy or ethical restrictions.
