# Supplementary material for: Efficacy of idecabtagene vicleucel in patients with relapsed/refractory multiple myeloma and prior central nervous system manifestation: A multicenter real‐world analysis
Source: Hemasphere. 2025 Aug 18;9(8):e70192. doi: 10.1002/hem3.70192 (PMC12358732; doi:10.1002/hem3.70192)
Supplement: Supplementary file 1 — Supporting Information. [file HEM3-9-e70192-s002.docx]

**Supplementary Information**

**Efficacy of Idecabtagene Vicleucel in Patients with Relapsed/Refractory Multiple Myeloma and Prior Central Nervous System Manifestation: A Multicenter Real-World Analysis**

**Supplemental Table S1. Clinical characteristics at ide-cel therapy.**

| **Parameters** | **MM-CNS pts (N= 10)** |
| --- | --- |
| **Median age at diagnosis, years** (range) | 55 (44-72) |
| **Age at ide-cel infusion** |  |
| <60 years | 4 |
| 60-69 years | 5 |
| ≥ 70 years | 1 |
| **Female** | 6 |
| **ECOG >2** | 4 |
| **Myeloma type** |  |
| IgG Kappa | 6 |
| IgG Lambda | 3 |
| IgA Kappa | 1 |
| **R-ISS stage at diagnosis** |  |
| I | 2 |
| II | 4 |
| III | 4 |
| **Cytogenetics** |  |
| 1q gain | 2 |
| High risk aberrations without 1q gain | 3 |
| Standard risk | 5 |
| **Median therapy lines number prior to ide-cel** (range) | 5 (2-8) |
| **Triple-refractory disease** | 9 |
| **Penta-refractory disease** | 7 |
| **Prior HDCT/ASCT** | 9 |
| **Prior alloSCT** | 2 |
| **CNS manifestations** |  |
| Parenchymal lesions | 5 |
| Leptomeningeal | 6 |
| **Symptomatic CNS myeloma** | 9 |
| **Diagnostics of CNS myeloma** |  |
| MRI brain *and* cCT | 2 |
| MRI brain and spine *and* cCT | 2 |
| Cranial CT only | 1 |
| MRI brain only | 1 |
| MRI brain and spine only | 2 |
| MRI brain *and* CSF | 2 |
| **Manifestation of CNS myeloma** |  |
| Immediately prior to  bridging therapy to ide-cel | 5 |
| In previous therapy lines | 5 |
| **Median time from apheresis to infusion, days** (range) | 60 (47-112) |
| **Median time from diagnosis-ide-cel, years** (range) | 5.6 (3.5-13.6) |
| *t(4;14), t(14;16), t(14;20), 17p del.  ide-cel= idecabtagene vicleucel; MM= multiple myeloma; CNS= central nervous system; Pts= patients; ECOG= Eastern Cooperative Oncology Group; R-ISS= Revised International Staging System; HDCT/ASCT= high-dose chemotherapy and autologous stem cell transplantation; alloSCT= allogeneic stem cell transplantation; MRI= magnetic resonance imaging; cCT= cranial computed tomography; CSF= cerebrospinal fluid. | |

**Supplemental Table S2.** **Treatment sequence and outcomes of MM patients with CNS disease before and after ide-cel.**

| **Patient** | **Therapy sequence** | **Site of CNS myeloma** | **Last therapy preceding MM-CNS** | **Symptoms** | **Treatment for CNS myeloma prior ide-cel** | **Best response to bridging therapy** | **Modality of CNS response evaluation after ide-cel** | **Best objective response** | **Outcomes** |
| --- | --- | --- | --- | --- | --- | --- | --- | --- | --- |
| 1 | 1L: 3x Vd 🡪 PR 🡪 HDCT/ASCT 🡪 VGPR  2L: alloSCT 🡪 VGPR, thalidomide maintenance, IF positive, 3x DLI 🡪 PD (osteolysis)  3L: Rd 🡪 PD serologically  4L: Pd 🡪 VGPR 🡪 PD 🡪RT  5L: DRd 🡪 PD, surgery  6L: Dara-Vd 🡪 daratumumab maintenance 🡪 new bone lesions  7L: Kd 🡪 PD with new CNS disease | Intradural | Kd | Pain and gait disturbance | IKEMA (4x)  + RT of spinal cord (8^th^ line) | CR serologic;  CNS lesion not assessed, clinical response with improvement of neurology | MRI | CR serologic + CR CNS | Alive in CR (radiologically +  serologically)  16 mo after ide-cel;  CNS myeloma vanished completely, no neurological symptoms |
| 2* | 1L: Surgery and RT (symptoms of paraplegia) 🡪 Rd 🡪 PD 🡪 new cranial bone lesions 🡪 RT  2L: 3x VCd 🡪 PR 🡪 HDCT/ASCT 🡪 serological CR for 22 mo 🡪 PD with new CNS disease | Intracerebral, paramedial left side | VCd followed by HDCT/  ASCT | Impaired vision,  dizziness | Cranial RT, dexamethasone, surgery (3^rd^ line);  Dara-Kd 🡪 PD 🡪 surgery 🡪 PD 🡪 Elo-Pd (bridging) | CR serologic + CR CNS | MRI | CR serologic + CR CNS | Alive in CR  12 mo after ide-cel therapy |
| 3 | 1L: 4x VCd 🡪 PR; 1^st^ HDCT/ASCT 🡪 VGPR 🡪 lenalidomide maintenance 🡪 serological PD  2L: 3x Dara-Vd 🡪 PR; 2^nd^ HDCT/ASCT 🡪 CR serologically for 18 mo 🡪 massive PD with fresh CNS disease | Extensive meningeosis myelomatosa with massive neurological deficit | 2^nd^ HDCT/ASCT | Aphasia,  Apraxia,  Epileptic seizures | 8x Dara-PVPD, 23x thiothepa/ dexamethasone i.th. (3^rd^ line) | CR serologic + PR CNS  (meningeosis myelomatosa with a few tumor cells only in CSF) | MRI + CSF | CNS imaging: response with residuals after meningeosis myelomatosa, CSF: CR;  CR serologic | Alive in CR of CNS disease and serologic CR 14 mo post ide-cel |
| 4* | 1L: 3x VCd 🡪 HDCT/ASCT 🡪 lenalidomide maintenance 🡪 PD with osteolysis  2L: RT, 1x Benda 🡪 mixed response  3L: Dara-Pd 🡪 VGPR, new bone lesions 🡪 RT 🡪 new CNS disease (intraspinal), signs of myelon damage in MRI | Intradural | Dara-Pd | Paraplegia, neurogenic  bowel/bladder dysfunction | Surgery, spinal RT + Elo-Pd  (4^th^ line) | PR serologic + PD CNS | MRI | PR serologic + PR CNS | Alive in serologic and CNS PR 6 mo post ide-cel at last FU |
| 5* | 1L: P-Vd 🡪 PD  2L: VRd 🡪 lenalidomide maintenance 🡪 PD  3L: Dara-Kd 🡪 PD 🡪 with new CNS disease | Intradural, orbita | Dara-Kd | Lumbago,  visual impairment | Craniospinal RT, cortisone, surgery, Elo-Pd (4^th^ line) | PD serologic + CR CNS | CT + MRI | CR serologic + CR CNS | Systemic relapse 1.5 mo post ide-cel; death from systemic non-CNS relapse/refractory disease 7 mo post CAR-T, CNS disease with maintained CR after ide-cel |
| 6 | 1L: VCd 🡪 HDCT/ASCT 🡪 PD with meningeosis myelomatosa | Meningeosis myelomatosa | VCd followed by HDCT/  ASCT | Headache,  vertigo | i.th. triple therapy (methotrexate/hydrocortisone/cytarabine) + MARiTA (2^nd^ line) and DeVIC (3^rd^ line)  🡪 2x Dara-Kd (bridging) | PR serologic + PD CNS | MRI | PR serologic + SD CNS | Systemic progress 9 mo and progress of CNS disease 12 months post CAR-T; death from r/r MM 13 mo after ide-cel with meningeosis myelomatosa |
| 7 | 1L: VCd 🡪 1^st^ HDCT/ASCT 🡪 lenalidomide maintenance 🡪 PD with new CNS manifestation | Meningeosis  myelomatosa | HDCT/ASCT, lenalidomide maintenance | Facial nerve palsy | i.th. triple therapy, cranial RT, 2x MATRix followed by 2nd HDCT/ASCT  (2^nd^ line) 🡪 Dara-PACE (bridging) | VGPR serologic + PR CNS | MRI | VGPR serologic + CR CNS | Systemic relapse 5 mo post ide-cel; died from PD 13 mo after CAR-T;  ongoing CR of CNS disease post ide-cel |
| 8* | 1L: Rd 🡪 PR 🡪 new bone lesions  2L: Kd 🡪 HDCT/ASCT 🡪 relapse  3L: DRd 🡪 PD  4L: Dara-Vd 🡪 PD  5L: alloSCT 🡪 CR 🡪 relapse  6L: Isa-Pd 🡪 PD with CNS manifestation | Meningeosis myelomatosa, spinal infiltration | Isa-Pd | Paraparesis of lower limbs | Craniospinal RT (7^th^ line) 🡪  Dara-Kd + selinexor (bridging) | VGPR serologic +  PR CNS in MRI, no myeloma cells in CSF | MRI + CSF | CR serologic + CR CNS | Alive in CR (serologic + CNS manifestation) 10 mo after CAR-T, recurrent infections |
| 9 | 1L: 3x E-KRd 🡪 RT 🡪 VGPR 🡪 HDCT/ASCT 🡪 VGPR 🡪 maintenance with elotuzumab and lenalidomide 🡪 PD  2L: Dara-Vd 🡪 PD  3L: Elo-Pd 🡪 PD  4L: Talquetamab 🡪 PR 🡪 PD with CNS disease | Intradural,  cerebral | Talquetamab | Paraplegia of lower limbs | Cranial RT + Dara-VDT-PACE (5^th^ line) + i.th. triple therapy | CR serologic + PR CNS | MRI + CSF | PD serologic + PD CNS | Refractory, died from massive systemic and CNS progress 4 mo after CAR-T |
| 10* | 1L: VRd 🡪 HDCT/ASCT 🡪 lenalidomide maintenance 🡪 relapse  2L: Dara-Pd 🡪 PD  3L: Dara-Vd 🡪 PD with CNS disease | Chiasmal compression | Dara-Vd | Bitemporal hemianopsia | Cranial RT with 30 Gy, later Elo-Rd (4^th^ line) 🡪 Isa-Kd (bridging) | PD serologic + PR CNS | MRI | PR serologic + PR CNS | Alive in PR (serological + CNS) 6 mo post ide-cel;  no neurological deficiency |

CNS, central nervous system; CAR-T, chimeric antigen receptor T-cell therapy; non-CNS, non-central nervous system; FU, follow-up; HDCT/ASCT, high-dose chemotherapy and autologous stem cell transplantation; PD, progressive disease; MATRix, methotrexate, rituximab, cytarabine, thiotepa; MARiTA (Rituximab, HD-MTX, AraC, cytarabine followed by HDCT/ASCT); DeVIC, carboplatin, dexamethasone, ifosfamide, etoposide; MRI, magnetic resonance imaging; CR, complete remission; PR, partial remission; VGPR, very good partial remission; RT, radiotherapy; Gy, gray; CT, computed tomography; Vd, bortezomib, dexamethasone; VRd, bortezomib, lenalidomide, dexamethasone; VCd, bortezomib, cyclophosphamide, dexamethasone; alloSCT, allogeneic stem cell transplantation; DLI, donor lymphocyte infusion; Rd, lenalidomide, dexamethasone; DRd, daratumumab, lenalidomide, dexamethasone; Dara-Vd, daratumumab, bortezomib, dexamethasone; Dara-Kd, daratumumab, carfilzomib, dexamethasone; Dara-Pd, daratumumab, pomalidomide, dexamethasone; Dara-VRd, daratumumab, bortezomib, lenalidomide, dexamethasone; Kd, carfilzomib, dexamethasone; Isa-Kd, isatuximab, carfilzomib, dexamethasone; Isa-Pd, isatuximab, pomalidomide, dexamethasone; Elo-Pd, elotuzumab, pomalidomide, dexamethasone; Elo-Rd, elotuzumab, lenalidomide, dexamethasone; Dara-PVPD, daratumumab, bortezomib, procarbazine, dexamethasone; PVd, pomalidomide, bortezomib, dexamethasone; Cd, cyclophosphamide, dexamethasone; i.th., intrathecal therapy; PACE, cisplatin, doxorubicin, cyclophosphamide, etoposide; VTd-PACE, bortezomib, thalidomide, dexamethasone, cisplatin, doxorubicin, cyclophosphamide, etoposide; IKEMA, isatuximab, carfilzomib, dexamethasone; IF, immunofixation; IEV, ifosfamide, epirubicin, etoposide; BSC, best supportive care; E-KRd, elotuzumab, carfilzomib, lenalidomide, dexamethasone; Pd, pomalidomide, dexamethasone; mo, months.

* The indicated patients had been diagnosed with CNS myeloma in previous therapy line

**Supplemental Table S3. Clinical characteristics for pair-matched cohort of non-CNS myeloma pts.**

|  | **Non CNS-MM  (N=24)** | **MM-CNS (N=8)** | **P-value** |
| --- | --- | --- | --- |
| **Median age at diagnosis, years** (range) | 55 (46-70) | 56 (44-67) | 0.916 |
| **Median age at ide-cel infusion, years** (range) | 63 (50-75) | 62 (48-72) | 0.688 |
| **ECOG at ide-cel** |  |  | 0.109 |
| ECOG 0-2 | 22 (100%) | 6 (85.7%) |  |
| ECOG >2 | 0 | 1 (14.3%) |  |
| Missing | 2 | 0 |  |
| **R-ISS Stage at Diagnosis** |  |  | 0.418 |
| I | 3 (14.3%) | 0 (0%) |  |
| II | 7 (33.3%) | 4 (66.7%) |  |
| III | 11 (52.4%) | 2 (33.3%) |  |
| Missing | 3 | 0 |  |
| **Median therapy lines prior to ide-cel** (range) | 5 (3-8) | 4.5 (3-8) | 0.724 |
| **Response status** |  |  | 1.000 |
| CR | 6 (25.0%) | 2 (25.0%) |  |
| VGPR | 3 (12.5%) | 1 (12.5%) |  |
| PR | 9 (37.5%) | 3 (37.5%) |  |
| nonPR | 6 (25.0%) | 2 (25.0%) |  |

CNS= central nervous system; MM= multiple myeloma; Pts= patients; ide-cel= idecabtagene vicleucel; ECOG= Eastern Cooperative Oncology Group; R-ISS= Revised International Staging System; CR= complete remission; VGPR= very good partial remission; PR= partial remission.

**Supplemental Table S4. Toxicities for pair-matched cohorts.**

|  | **Non CNS-MM  (N=24)** | **MM-CNS (N=8)** | **P-value** |
| --- | --- | --- | --- |
| **CRS** |  |  |  |
| None | 3 (12.5%) | 1 (12.5%) | 1 |
| Grade 1 | 11 (45.8%) | 3 (37.5%) | 0.692 |
| Grade 2 | 5 (20.8%) | 2 (25%) | 0.406 |
| Grade 3 | 5 (20.8%) | 2 (25%) | 0.406 |
| Grade 4 | 0 | 0 | - |
| **ICANS** |  |  |  |
| None | 18 (75%) | 7 (87.5%) | 0.475 |
| Grade 1 | 4 (16.7%) | 1 (12.5%) | 0.787 |
| Grade 2 | 2 (8.3%) | 0 | 0.415 |
| Grade 3 | 0 | 0 | 1 |
| Grade 4 | 0 | 0 | 1 |
| **HLH** |  |  |  |
| Yes | 0 | 0 | 1 |
| No | 24 (100%) | 8 (100%) |  |
| **Tocilizumab use** |  |  |  |
| Yes | 15 (62.5%) | 7 (87.5%) | 0.198 |
| No | 9 (37.5%) | 1 (12.5%) |  |
| **Steroid use** |  |  |  |
| Yes | 4 (16.7%) | 3 (37.5%) | 0.230 |
| No | 20 (83.3%) | 5 (62.5%) |  |
| **Anakinra use** |  |  |  |
| Yes | 0 | 0 | 1 |
| No | 24 | 8 (100%) |  |
| **Delayed Parkinsonian neurotoxicity** |  |  |  |
| Yes | 0 | 0 | 1 |
| No | 24 | 8 (100%) |  |
| **Delayed non-Parkinsonian neurotoxicity** |  |  |  |
| Yes | 0 | 0 | 1 |
| No | 24 | 8 (100%) |  |
| **Infections** |  |  |  |
| Yes | 16 (66.7%) | 6 (75%) | 0.535 |
| No | 8 (33.3%) | 2 (25%) |  |

CNS= central nervous system; MM= multiple myeloma; CRS= cytokine release syndrome; ICANS= immune effector cell-associated neurotoxicity syndrome; HLH= Hemophagocytic lymphohistiocytosis.
