# Supplementary material for: Efficacy of idecabtagene vicleucel in patients with relapsed/refractory multiple myeloma and prior central nervous system manifestation: A multicenter real‐world analysis
Source: Hemasphere. 2025 Aug 18;9(8):e70192. doi: 10.1002/hem3.70192 (PMC12358732; doi:10.1002/hem3.70192)

Supplemental Figure 1. CNS and serologic response of patient 8 showing complete remission after ide-cel.

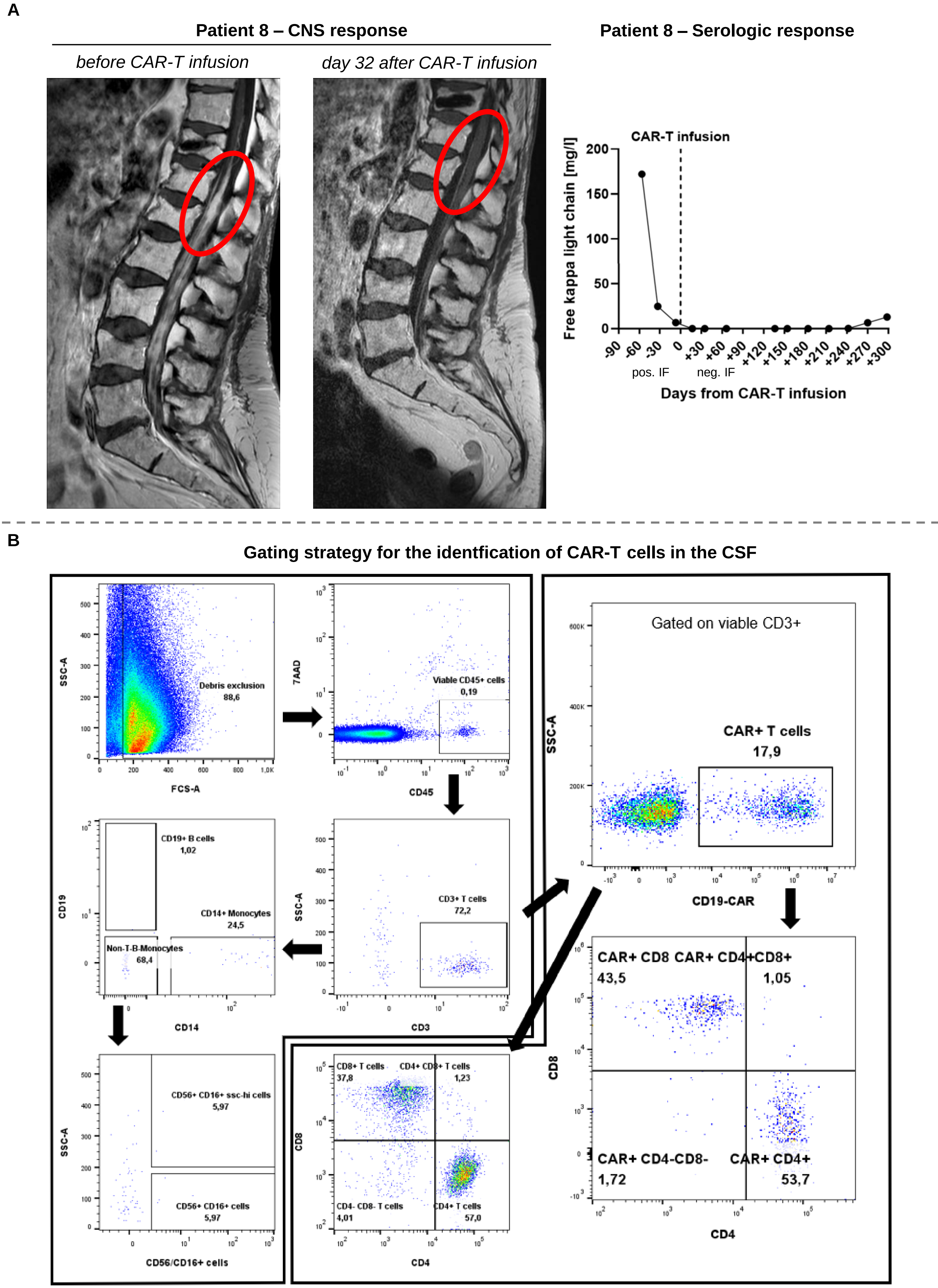

Supplement: Supplementary file 2 — Supporting Information. [file HEM3-9-e70192-s001.pdf]
